# Anesthetic Management of Tracheal Resection and Anastomosis for Postintubation Tracheal Stenosis Involving a Sequential Airway Strategy

**DOI:** 10.1155/cria/5633253

**Published:** 2026-08-30

**Authors:** Mohammad Zishan Uddin, Maliha Karim, Md. Abdul Karim, Md. Abdullah-Al-Mahammud Kabir, Hasib Ahamed, Md. Shahinur Rahman, Niaz Ahmed

**Affiliations:** ^1^ Department of Cardiothoracic-Vascular Anesthesia & ICU, Evercare Hospital, Dhaka, Bangladesh; ^2^ Department of Anesthesia, Analgesia & ICU, Sir Salimullah Medical College Hospital, Dhaka, Bangladesh; ^3^ Department of Cardiothoracic Anesthesia & ICU, Labaid Cardiac Hospital, Dhaka, Bangladesh; ^4^ Department of Thoracic Surgery, Evercare Hospital, Dhaka, Bangladesh

**Keywords:** bilateral superficial cervical plexus block, cross-field ventilation, postintubation tracheal stenosis, shared airway, tracheal resection and anastomosis

## Abstract

A postintubation tracheal stenotic patient who requires surgical resection and anastomosis is a challenging case for an anesthesiologist, requiring meticulous planning during surgical correction due to a shared airway. This case report is about the anesthetic management of a 23‐year‐old male who developed severe tracheal stenosis following prolonged mechanical ventilation after decompressive craniectomy and presented with severe dyspnea, wheeze, and stridor five weeks later. Tracheal resection and anastomosis were the ultimate solution for this patient. For that, a sequential airway strategy was used. This strategy includes distal tracheal intubation under bilateral superficial cervical plexus block, along with conscious sedation, followed by general anesthesia with cross‐field ventilation, bronchoscopy‐guided resection, and intermittent apneic technique during anastomosis. The patient was extubated at the table, and total recovery was uneventful. This case demonstrates the successful application of a sequential airway strategy, which facilitates safe tracheal surgery and immediate extubation.

**Learning points**
• Tracheal stenosis requiring resection and anastomosis poses a significant anesthetic challenge.
• A sequential airway strategy can maintain oxygenation, ventilation, and operative visibility, resulting in an optimal outcome.
• Multidisciplinary communication is essential.
• Integration of regional anesthesia in airway surgery improves overall intraoperative and postoperative outcomes.
• Selected patients may be safely extubated on the table.

## 1. Introduction

Severe postintubation tracheal stenosis is a rare but grave complication of prolonged endotracheal intubation (ETT). Its incidence is about 10%–22%, but among them, 1%‐2% develop severely symptomatic stenosis [1]. It may occur at any part of the trachea. However, the most common site is the endotracheal tube cuff contact site within the tracheal lumen [2], due to impairment of blood flow in the tracheal wall exerted by excessive ETT cuff pressure, resulting in ischemia [3], and may lead to fibrosis within 3–6 weeks [4]. Clinical features include exertional dyspnea, inspiratory stridor, and dyspnea at rest, depending on the degree of intraluminal tracheal narrowing [5]. Surgical resection and anastomosis are the definitive treatment for symptomatic patients with severe stenosis [6], but anesthetic management is challenging due to a shared airway and loss of airway continuity. The operative mortality rate is about 3% in the dedicated center, and the overall operative mortality rate is about 7%–11% [5]. We describe a case that highlights the implementation of a sequential airway strategy, which enables the safe conduct of anesthesia during tracheal resection and anastomosis.

## 2. Case Description

Written informed consent was obtained from the patient for publication of this case report, prepared in accordance with the CARE guidelines of the EQUATOR Network.

### 2.1. Patient Information

A 23‐year‐old male suffered a road traffic accident followed by blunt trauma to the left temporal lobe and developed intracranial hemorrhage requiring decompressive craniectomy. During his stay at the intensive care unit (ICU), he developed pneumonia, required mechanical ventilation, and remained intubated for 8 days and was later extubated. One month later, left‐side cranioplasty with titanium‐coated mesh was done under general anesthesia, and the patient remained on mechanical ventilation for one day and then was extubated. He developed dyspnea and stridor, which progressively worsened over the subsequent 5 weeks of decompressive craniectomy, and was readmitted to the hospital.

### 2.2. Clinical Findings

The patient presented with severe exertional dyspnea and bilateral wheeze with both inspiratory and expiratory stridor. Room air oxygen saturation was 94%–96%. The patient had frequent episodic attacks of paroxysmal dyspnea followed by cessation of breathing for a few seconds.

### 2.3. Diagnostic Assessment

**FIGURE 1:**
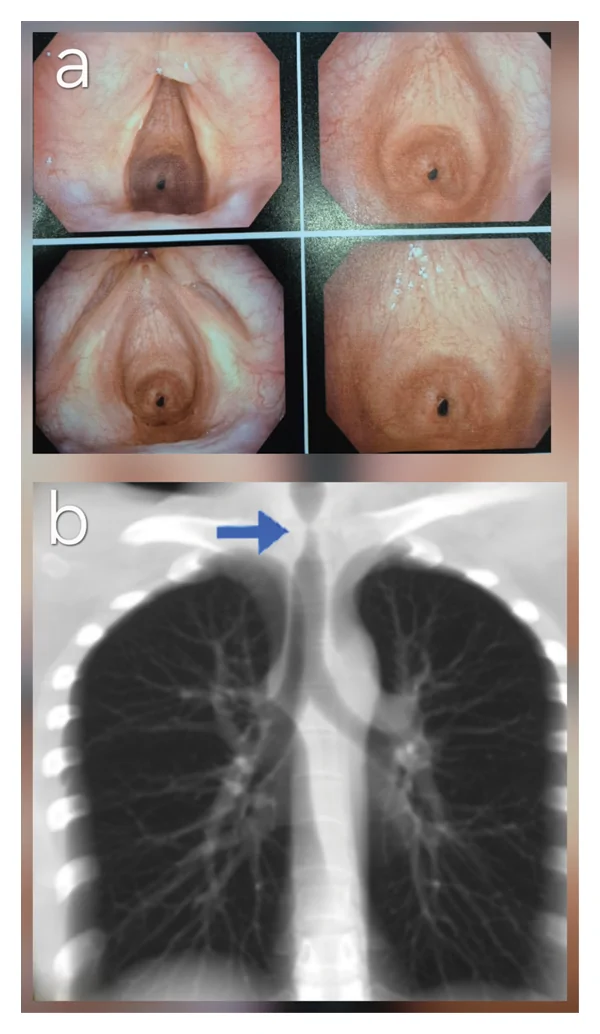
(a) Fiberoptic laryngoscopy and (b) high‐resolution CT of neck and thorax with three‐dimensional reconstruction of the airway showing tracheal stenosis in cervical trachea.

Flexible fiberoptic laryngoscopy (Figure 1a) and CT scan of the neck (Figure 1b) revealed severe tracheal stenosis (Grade 3) just below the 3.5‐cm level of the vocal cord, measuring about 11 mm in length. The lesion was classified as Myer–Cotton Grade 3 because the bronchoscope could not be advanced through the stenotic segment, and functional luminal obstruction was estimated to exceed 70%, while a residual lumen remained. Although originally developed for subglottic stenosis, the classification was used descriptively to communicate the severity of airway narrowing in this case.

### 2.4. Timeline

Table 1 describes the clinical course and events.

**TABLE 1:** Clinical timeline.

Time point	Event
Day 0	Road traffic accident; emergency decompressive craniectomy
Days 1–8	Mechanical ventilation in the ICU
Day 8	Extubation
1 month postinjury	Cranioplasty under general anesthesia
5 weeks postcraniectomy	Progressive dyspnea and inspiratory stridor
∼2.5 months postcraniectomy	Tracheal resection and end‐to‐end anastomosis
Postoperative day 0	Extubation on the table
Postoperative day 1	ICU observation
Postoperative days 2–8	Shifted and remained in the ward
Postoperative day 9	Discharged from the hospital

### 2.5. Therapeutic Intervention

The tracheal resection and anastomosis were planned. On the scheduled day, the patient was brought to the procedure room. Ultrasound‐guided radial artery cannulation was done for hemodynamic monitoring and for repeated arterial blood gas (ABG) sampling, if needed. An ultrasound‐guided femoral central venous catheter was inserted to provide central venous access remote from the surgical field and to facilitate rapid escalation should extracorporeal support become necessary. The patient also had fragile veins with a prior history of difficult intravenous cannulation. A nasogastric tube was inserted to identify the esophagus during tracheal dissection.

Ultrasound‐guided bilateral superficial cervical plexus block (SCPB) was performed for intraoperative distal tracheal intubation under spontaneous ventilation to reduce airway manipulation and provide adequate postoperative analgesia. Only bupivacaine 0.5% (75 mg) was used as a local anesthetic agent, combined with dexamethasone (5 mg). The total volume of the local anesthetic solution was 16 mL, 8 mL on each side. Dexamethasone was used to prolong the duration of analgesia. The patient was then transferred to the operating theater.

Conscious sedation was maintained with a continuous remifentanil infusion and incremental propofol. The initial propofol dose was 0.5 mg/kg, followed by a 10‐mg bolus as needed. Remifentanil was initiated at 0.2 μg/kg/min and continuously titrated according to respiratory pattern, bispectral index (BIS), end‐tidal carbon dioxide (EtCO_2_), and Ramsay sedation score while preserving spontaneous ventilation. Airway rescue equipment and an experienced multidisciplinary airway team remained immediately available throughout this phase. BIS level was maintained around 80, and Ramsay sedation scale 3 was maintained. BIS monitoring was used as an adjunct to clinical assessment; however, BIS values have not been fully validated for conscious sedation levels.

**FIGURE 2:**
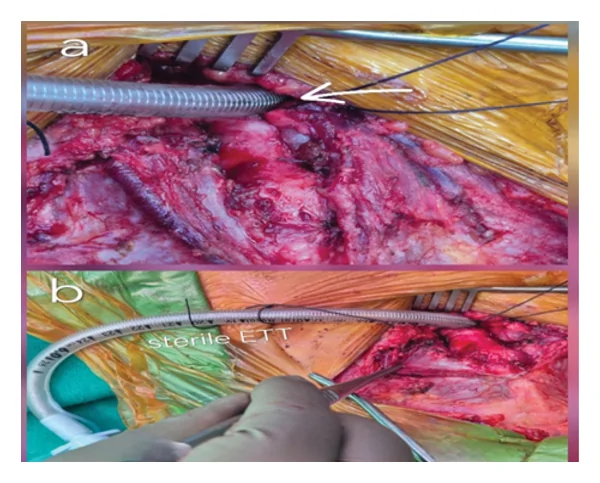
(a) Distal tracheal intubation. (b) Cross‐field ventilation.

Under bilateral SCPB and conscious sedation while maintaining spontaneous ventilation, the surgeon first exposed the cervical trachea distal to the stenotic segment. Following surgical exposure, a 6‐mm reinforced ETT was inserted directly into the surgically exposed distal trachea through the operative field, and cross‐field ventilation was then established (Figures 2a,b). After confirmation of placement of the armored ETT, a nondepolarizing muscle relaxant (vecuronium) was given. Then, the patient was maintained with total intravenous anesthesia (TIVA) with propofol (0.2 mg/kg/min) and remifentanil (0.5 μg/kg/min) with positive‐pressure ventilation. Tidal volume was set to 400–450 mL, respiratory rate was 14–18 per minute, and peak airway pressures remained 25–30 cm of H_2_O. Ventilator settings were adjusted to maintain adequate gas exchange.

**FIGURE 3:**
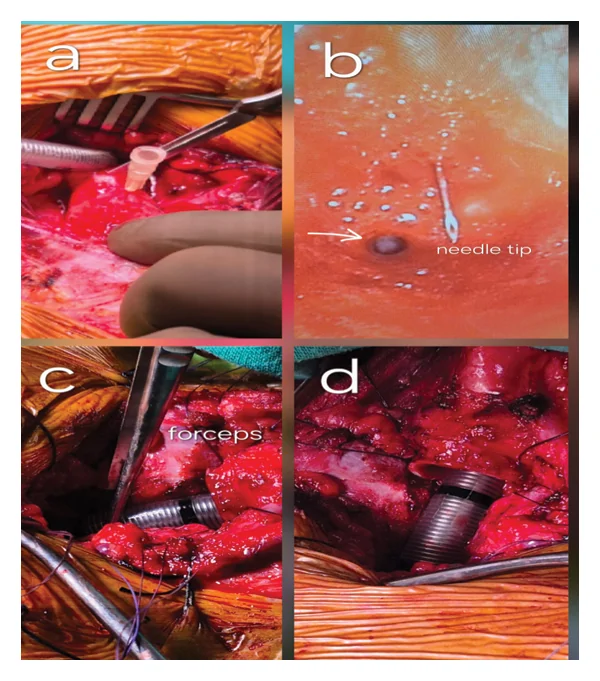
(a) A small‐bore needle inserted in the airway guided by the flexible bronchoscopy light. (b) Bronchoscopy view of tracheal stenosis with needle localization technique to differentiate between the healthy and granulation tissue. (c) Passing an endotracheal tube from the proximal tracheal stump to the distal tracheal stump by using forceps and (d) well placed beyond both resected tracheal stumps.

Fiberoptic bronchoscopy was used to identify the upper limit of tracheal stenosis and to differentiate between healthy and granulation tissue, guided by the needle localization technique (Figures 3a,b). Though preoperative CT estimated the lesion to be approximately 3.5 cm below the vocal cord, intraoperative bronchoscopy and surgical assessment localized the stenotic segment primarily to the second and third tracheal rings. The distal end of tracheal stenosis was identified by careful exposure of the cervical trachea. Then both the second and third tracheal stenotic rings were excised. The length of the resected tracheal segment was 1.2 cm.

Using a King Vision video laryngoscope, a 6‐mm cuffed ETT was inserted orally. DeBakey forceps were used to pass the ETT from the proximal tracheal stump to the distal tracheal stump, through the operative field (Figures 3c,d). Simultaneously, the distal armored ETT was removed. The patient was then ventilated with 100% oxygen for 5 min before the tube was withdrawn for suture placement. Using the apneic technique, the posterior anastomosis was performed within 4 min. Then, the tube was reinserted similarly, kept proximally to the anastomosis site, and ventilation was resumed with 100% oxygen for 5 min. The lowest recorded SpO_2_ was 91%, recovering to 99% within 30 s after reinstitution of ventilation. EtCO_2_ before and after the apneic phase was 34 and 46 mmHg, respectively. Then, the anterior anastomosis was completed over the ETT. Bag‐mask ventilation was performed at 30 cmH_2_O, while the anastomosis was under saline, and there was no air leak.

A guardian chin stitch was performed to limit neck extension. It not only reduced the anastomotic tension but also reduced the risk of anastomotic leak, dehiscence, or restenosis. Throughout the time, the patient remained hemodynamically stable, and all vital parameters remained within normal limits. ETT cuff pressures were monitored and maintained below 25 cmH_2_O.

### 2.6. Follow‐Up and Outcomes

Deep extubation was done on the table after confirming adequate spontaneous ventilation, full neuromuscular recovery, and absence of airway edema. There was no gagging, coughing, or spasm. An ABG was done just after shifting the patient to the ICU and demonstrated satisfactory oxygenation and ventilation (pH 7.41, PaO_2_ 110 mmHg with 5 L/min of O_2_, and PaCO_2_ 37 mmHg). The patient was observed in the ICU for 24 h and had an uneventful recovery. A bilateral SCPB provided analgesia for 24 h, and later, the patient was managed only with oral paracetamol. No opioid was needed in the postoperative period. The patient was relieved of symptoms and was overall immensely satisfied with the process. The guardian suture was removed on the sixth postoperative day, and the patient was discharged on the ninth postoperative day. At 6‐week follow‐up, the patient remained asymptomatic without stridor or exertional dyspnea.

## 3. Discussion

Symptomatic severe tracheal stenosis requires surgical resection and anastomosis, and the overall success rate is more than 90% [7]. It requires close collaboration between the anesthesia and surgical team for a successful outcome due to shared airway, and it is challenging for the anesthesiologists to maintain adequate ventilation and oxygenation and avoid complications like laryngospasm, aspiration, surgical fire, and vocal cord paralysis [8].

SCPB provides anesthesia to the skin and superficial tissues of the anterior triangle of the neck by blocking anterior primary rami of the two to four cervical nerves. It provides excellent postoperative analgesia [9]. There are few reports of SCPB‐assisted spontaneous breathing techniques in airway surgery [10–12]. For this patient, bilateral SCPB anesthetized the skin and superficial tissues of the anterior neck, facilitating cervical incision and tracheal exposure while preserving spontaneous ventilation. However, SCPB does not anesthetize the tracheal mucosa or recurrent laryngeal nerve territory. Airway tolerance during distal tracheal tube placement was achieved through carefully titrated conscious sedation together with placement of the tube directly into the surgically exposed distal trachea rather than by airway anesthesia from the block itself. This approach facilitated safe distal tracheal intubation while avoiding positive‐pressure ventilation before securing the airway distal to the stenosis. Potential complications of this block include inadvertent phrenic nerve involvement, local anesthetic systemic toxicity, vascular puncture, and hematoma formation. These risks were minimized by ultrasound guidance, careful aspiration before injection, and use of a limited volume of local anesthetic. As dexamethasone was added, it increased the duration of analgesia [13] and provided excellent postoperative analgesia. The patient remained totally opioid free in the postoperative period, thereby ensuring avoidance of opioid related complications.

The addition of conscious sedation improved patient comfort and reduced complications by maintaining a patent airway [14] before confirmation of distal tracheal intubation and initiation of cross‐field ventilation.

Controlled cross‐field ventilation provides a better operative field by connecting the distal reinforced ETT to the anesthesia machine via a sterile anesthetic circuit over a surgical drape. There are other ventilation options during the open‐airway phase. These include jet ventilation, cardiopulmonary bypass, and extracorporeal oxygenation. During jet ventilation, under (15–20 PSI) pressure, intermittent jets of inspiratory flow are provided by a narrow‐orifice catheter, but expiration is passive. Though it provides a better surgical visual field, it is challenging to monitor ventilation, with the risk of barotrauma and mucosal injury. Extracorporeal circulatory support is the ultimate salvage technique, which provides the best surgical field, gas exchange, and hemodynamic stability. However, it is associated with significant complications, which include coagulopathy, hemodilution, risk of vascular injury, prolonged mechanical ventilation, and lengthy stay at the ICU [5, 7, 15]. In this patient, regional anesthesia facilitated smooth distal tracheal intubation during spontaneous ventilation. After distal tracheal intubation, a muscle relaxant was applied, and cross‐field ventilation was initiated.

Intraoperative bronchoscopy is an important tool used not only to diagnose the exact location of stenosis but also to differentiate between healthy and granulation tissue, thereby helping to guide precision‐based resection [5, 7].

TIVA was chosen instead of an inhalational agent, which ensured adequate delivery of an anesthetic agent, especially during the open airway phase. The fraction of inspired oxygen (FiO2) was kept around 30% to avoid fire risk due to the open trachea. However, the patient was ventilated with 100% oxygen for 5 min before and after the apneic technique to prevent desaturation [16]. Electrocautery was not active during high‐FiO_2_ phases to minimize airway fire risk. Only one episode of desaturation occurred at the end of posterior anastomosis, which was managed by manually ventilating 100% oxygen after reintroduction and placement of the ETT beyond the posterior anastomosis.

Deep extubation ensures a smooth emergence from anesthesia by reducing the risk of coughing and bucking at the fresh anastomosis, made possible by good postoperative analgesia from the regional block. This approach attenuated the extubation reflex, facilitating a smooth and hemodynamically stable extubation. It requires cautious monitoring to avoid complications such as airway obstruction, laryngospasm, bronchospasm, aspiration, and oxygen desaturation [17]. The deep extubation technique not only enabled a smooth and uneventful recovery but also reduced significant tension on the fresh anastomotic suture in this patient.

Although bilateral SCPB‐assisted spontaneous breathing techniques have previously been described for airway surgery, including the report by Cho et al. [10], our case extends this concept by integrating SCPB into a complete sequential airway strategy consisting of surgical exposure under spontaneous ventilation, distal cross‐field tracheal intubation, controlled cross‐field ventilation, intermittent apneic anastomosis, and immediate opioid‐free table extubation. Rather than introducing a new individual technique, this case demonstrates how established airway and regional anesthesia techniques can be combined into a structured sequential approach for the management of severe postintubation tracheal stenosis.

## 4. Conclusion

This case highlights the critical role of systematic airway management planning and effective collaboration between anesthesia and surgical teams in achieving successful outcomes in tracheal surgery.

## Author Contributions

Mohammad Zishan Uddin: writing–original draft, visualization, project administration, methodology, intraoperative anesthetic management, data curation, and conceptualization.

Maliha Karim: writing–original draft, data curation, and literature review.

Md. Abdul Karim: writing–review and editing, validation, and conceptualization.

Md. Abdullah‐Al‐Mahammud Kabir: writing–review and editing and visualization.

Hasib Ahamed: writing–review and editing and visualization.

Md. Shahinur Rahman: writing–review and editing and validation.

Niaz Ahmed: writing–review and editing, validation, and supervision.

## Funding

No funding was received for this work.

## Consent

Consent was obtained for the use of the image, clinical information, and publications.

## Conflicts of Interest

The authors declare no conflicts of interest.

## Patient Perspective

The patient was satisfied with the overall management.

## Supporting Information

Additional supporting information can be found online in the Supporting Information section.

## Supporting information


**Supporting Information** CARE Checklist

## Data Availability

Data are available on request due to privacy/ethical restrictions.
