# Supplementary material for: Anesthetic Management of Tracheal Resection and Anastomosis for Postintubation Tracheal Stenosis Involving a Sequential Airway Strategy
Source: Case Rep Anesthesiol. 2026 Aug 30;2026:5633253. doi: 10.1155/cria/5633253 (PMC13526281; doi:10.1155/cria/5633253)
Supplement: Supplementary file 1 [file CRIA-2026-5633253-s001.docx]

# CARE Checklist

Manuscript Title: Anesthetic Management of Tracheal Resection and Anastomosis for Post-Intubation Tracheal Stenosis Involving a Sequential Airway Strategy

| CARE Item | Description | Reported in Manuscript | Location in the manuscript |
| --- | --- | --- | --- |
| Title | Diagnosis/intervention identified as a case report | Yes | Title (Page 1) |
| Keywords | 2–5 key words including 'case report' | Yes | Keywords (page 5) |
| Abstract | Background, case presentation, intervention, outcome, conclusion | Yes | Abstract (page 5) |
| Introduction | Brief background and rationale | Yes | Introduction (Page 6) |
| Patient information | Demographics, symptoms, history | Yes | Patient information (Page 6) |
| Clinical findings | Relevant examination findings | Yes | Clinical findings (Page 6) |
| Timeline | Chronological timeline of events | Yes | Clinical timeline (Page 7,8) & separate file |
| Diagnostic assessment | Investigations, imaging, diagnosis | Yes | Diagnostic assessment (Page 7) |
| Therapeutic intervention | Anesthetic and surgical management | Yes | Therapeutic intervention (Page 8,9,10) |
| Follow-up and outcomes | Postoperative course and follow-up | Yes | Follow-up and outcomes (Page 10) |
| Discussion | Strengths, limitations, literature comparison | Yes | Discussion (Page 10,11,12) |
| Patient perspective | Patient perspective included | Yes | Patient perspective (Page 13) |
| Informed consent | Written informed consent obtained | Yes | Case description (Page 6) |
